# Prospective study to evaluate quality of life in amyotrophic lateral sclerosis

**DOI:** 10.1038/s41598-023-39147-w

**Published:** 2023-07-26

**Authors:** Candela Caballero-Eraso, Carlos Carrera-Cueva, Esther de Benito Zorrero, Cecilia Lopez-Ramirez, Samira Marin-Romero, Maria Isabel Asensio-Cruz, Emilia Barrot-Cortes, Luis Jara-Palomares

**Affiliations:** 1 Medical Surgical Unit of Respiratory Diseases, Hospital Virgen del Rocío, Av. Manuel Siurot S/N, 41013 Seville, Spain; 2 Centro de Investigación Biomédica en Red de Enfermedades Respiratorias (CIBERES), Carlos III Health Institute, Madrid, Spain

**Keywords:** Amyotrophic lateral sclerosis, Neuromuscular disease

## Abstract

Amyotrophic lateral sclerosis (ALS) is a neurodegenerative rare disease characterized by symptoms and signs in the upper and lower motor neurons, leading to progressive neuro-degeneration and muscle atrophy. Our objective was to analyse the quality of life (QoL) in patients with ALS and compare with general population and with patients with cancer. Prospective study from consecutive ALS patients in one center. In order to assess quality of life, during the first visit three questionnaires were administered: Amyotrophic Lateral Sclerosis Functional Rating Scale (ALSFRS-R), Short Form-36 (SF-36) and EuroQoL 5D (EQ-5D). We compared SF-36 of ALS patients with a reference population (n = 9151), and we compared the EQ-5D index score of ALS patients versus patients with cancer in the same area and in the same period (2015–2018). Between June 2015 and September 2017, 23 were included. The mean age was 65.1 ± 12.6 years and 56.5% were women. Compared with the general population, patients with ALS showed lowest QoL (p < 0.05) in all the dimensions, with a very important impairment in physical function (median: 0; p25-75: 0–10) and physical role (median: 0; p25-75: 0–6.25). In EQ-5D questionnaire, patients with ALS presented an EQ-5D index score of 0.21 ± 0.39 (mean ± standard deviation) with a visual analog scale (VAS) score of 0.32 ± 0.24. Compared with an oncological population, patients with ALS had a worse EQ-5D index score both clinically and statistically (0.21 ± 0.39 vs. 0.77 ± 0.27; p < 0.05). We demonstrate a poorer quality of life in patients with ALS is poor, and clinically and statistically worse than in patients with cancer or general population. New studies need to evaluate the impact of strategies in this population to improve the quality of life.

## Introduction

Amyotrophic lateral sclerosis (ALS) is a rare neurodegenerative disease with a worldwide incidence of 0.6 and 3.8 per 100, 000 person-years^1, 2^. ALS is characterized by symptoms and signs of the upper and lower motor neurons, leading to progressive neuro-degeneration and muscle atrophy^3^. This disease also has a huge impact in the quality of life (QoL) of patients and caregivers^4–6^. Nowadays, one of the aims in patients with ALS is to improve the QoL. The QoL questionnaires are tools that quantify the health-related quality of life. These questionnaires can be generic or specific for a disease and require adaptation and validation. ALS affects the QoL of patients, although the real knowledge of the impact of the disease could be better interpreted if we compare their results with general population and with other well-known diseases. For that reason, we aimed to analyse the QoL in patients with ALS and compare with general population and with patients with cancer.

## Material and methods

This is a secondary analysis from a prospective study that evaluated the incidence of venous thromboembolism in ALS patients. The rationale, design and main results of the study were published previously^7^. The study was approved by the Ethical Committee of the center according to the Spanish Regulatory Authorities (0795-N-14), and each patient provided written informed consent. This work was conducted as per the principles of the Declaration of Helsinki and ICH Guidelines for Good Clinical Practice. All study documents were prepared according to Good Clinical Practice guidelines (CPMP/ICH/135/95). Individual data elements were purposely obtained for this study, anonymized and protected according to the European Union directive 2016/679 of the European Parliament and the European Council, of April 27, 2016. Briefly, consecutive patients with ALS were evaluated followed every 3 months until completing 2 years of follow up. Demographic data and comorbidities were recorded. The objective of this study was to evaluate the QoL in patients with ALS and compare QoL between ALS with two cohort (patients with cancer and general population).

Consecutive patients with ALS according to El Escorial criteria (clinically definite or probable ALS)^8^, with age > 18 years and with no previous anticoagulant treatment or with previous venous thromboembolism were enrolled from June 2015 to September 2017. In order to assess quality of life, during the first visit, three questionnaires were administered (caregivers helped patients to fill it in when necessary): Amyotrophic Lateral Sclerosis Functional Rating Scale (ALSFRS-R)^9^, Short Form-36 (SF-36)^10^ and EuroQoL 5D (EQ-5D)^11, 12^. ALSFRS-R is a specific questionnaire for ALS validated in Spanish in 2010^9^. This scale is an instrument to measure the progression of the disease in patients with ALS and that assesses the degree of patient´s immobility, the existence and degree of hypersalivation and bulbar involvement where lower scores indicate poorer function. The questionnaires SF-36^10^ and EQ-5D^11, 12^ are two generic quality-of-life questionnaires which will allow us to compare the ALS QoL with other groups. SF-36 scale, it’s available and validated in Spanish and reference values are available from the general population^10^. This questionnaire evaluates 36 items with 8 different dimensions: physical situation, physical role, body pain, general health, vitality, social function, emotional role, and mental health. EQ-5D is a health questionnaire develop by the EuroQol Group which allow to compare different diseases^11, 12^. This questionnaire allows the calculation of a utility index (UI), which comprises 5 different areas (mobility, self-care, daily activities, pain/discomfort, and anxiety/depression), with a score between 1 (without problem) to 5 (unable to/extreme problems). The responses are combined to obtain a 5-digit number (from 11,111 to 55,555) that summarizes the patient’s health status. This value is subsequently converted into the UI, which may range from 0.208 (worst possible health) to 1.000 (best possible health). The minimally important difference (MID) on the EQ-5D questionnaire for patients with malignancies is 0.06–0.08 UI^13^.

### Statistical analysis

For each QoL questionnaire, the distributions of scores, means, medians, range and standard deviation are presented. In this study, SF-36 questionnaire score was compared with a reference population using a population study performed in 9151 persons older than 18 years old^10^. Due to the social and media impact of cancer, we decided to compare the EQ-5D index score of ALS patients with patients with cancer (n = 249), taking reference to the work made in the same hospital and same period (2015–2018) by our group^14^. Statistical significance was considered when p value was < 0.05. Box Plots depicts the median (black line inside the box) and the interquartile range (the edges of the box). Statistical calculations were performed using SPSS, version 20.0 (SPSS Inc. 233 South Wacker Drive, 11th Floor Chicago, IL).

### Ethics approval

Study protocol has been approved by the Ethics Committee of Virgen del Rocio Hospital, authorization number 0795-N-14, for protection of human beings involved in biomedical research. All patients provided written consent.

## Results

Between June 2015 and September 2017, 44 patients were evaluated, of whom 23 were included in the analysis of QoL. Comparison between clinical characteristics of patients who were included vs. not included is in eTable Supplement 1. In brief, patients who did not participate in the study had similar characteristics although they were younger, with more non-invasive ventilation, supplemental oxygen, cough assist and reduce mobility and wheelchair.

Of 23 patients, mean age was 65.1 ± 12.6 years and 56.5% were women. The median duration of symptom until evaluation was 32.4 months (p25–75: 10.3–47). More than half (60.9%) had reduced mobility and 34.8% needed wheelchair. Out of all the patients studied, 8 presented both spinal and bulbar involvement, while 15 had only spinal involvement (3 of them presenting as flail arms). Two patients had familial ALS, and 19 had sporadic ALS. The functional and clinical status measured by ALSFRS-R questionnaire showed a median of 36 points (p25–p75: 28–38.75) (Table 1). Patients (n = 15) received a disability pension, with the distribution as follows: less than 800 euros per month (n = 1), 800–1500 euros per month (n = 3), and more than 1500 euros per month (n = 11). We obtained data about caregivers for 14 patients, and the relationships were as follows: husband and children (n = 3), wife and children (n = 3), wife (n = 3), nursing home (n = 2), husband (n = 1), children (n = 1), and mother (n = 1). Of all the patients evaluated in our unit, 10 patients (43.5%) were referred to the mental health unit for assessment of anxiety and/or depression. Before being evaluated by the mental health unit, only 4 patients (17.4%) were taking treatment for anxiety and/or depression, and after being evaluated, 11 patients (47.8%) received treatment.

**Table 1:** Baseline demographics and clinical features of patients with amyotrophic lateral sclerosis.

	Total (n = 23)
Men, n (%)	10 (43.5)
Age (years), mean ± DE	65.1 ± 12.6
Current smoker, n (%)	6 (26.1)
Familiar ALS, n (%)	2 (8.7)
Symptoms	
Bulbar, n (%)	10 (43.5)
Dysphagia, n (%)	10 (43.5)
Sialorrhea, n (%)	2 (9.5)
Dyspnoea, n (%)	7 (30.4)
Orthopnoea, n (%)	2 (13)
Headache, n (%)	3 (13)
Hypersomnolence, n (%)	4 (17.4)
Reduced mobility, n (%)	14 (60.9)
Use of wheelchair, n (%)	8 (34.8)
ALSFR, median (p25-075)	36 (28–38.75)
Non-invasive ventilation, n (%)	6 (26.1)
Tracheostomy, n (%)	0
Physiotherapy, n (%)	18 (78.3)
Inspirometer, n (%)	18 (78.3)
Manual assisted cough, n (%)	8 (34.8)
Mechanical Assist cough, n (%)	0 (0)
Oxygen therapy, n (%)	2 (9.1)
Percutaneous gastrostomy, n (%)	3 (13)
First evaluation in respiratory unit	
FVC (%), mean ± DE	92 ± 19.3
pCO_2_ (mmHg), mean ± DE	39.9 ± 4.7
pO_2_ (mmHg), mean ± DE	77.1 ± 10
Initial peak flow, mean ± DE	305.5 ± 121
At the start of non-invasive ventilation	
FVC (%), mean ± DE	52.3 ± 6.1
pCO_2_ (mmHg), mean ± DE	52 ± 7.1
pO_2_ (mmHg), mean ± DE	77.1 ± 10
HCO_3_ (mmol/l), mean ± DE	29.1 ± 5.5

**Figure 1:**
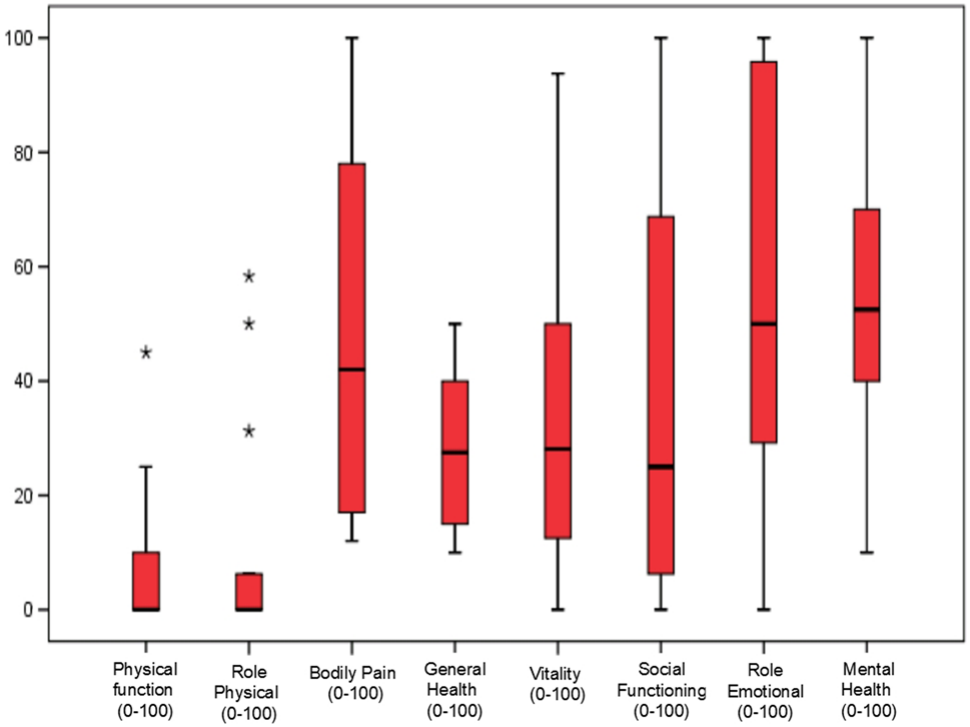
Box Plots of SF-36 in patients with amyotrophic lateral sclerosis. The bottom and the top of the box represent first and third quartile, respectively. The line in the center of the box is the median. The extreme of the whisker represents minimum and maximum. Outliers are plotted as individual asterisk.

Regarding the SF-36 questionnaire, the median of the physical and mental component was 23.3 (range: 10.7) and 36.5 (range: 51.63), respectively (Fig. 1). When compared with the general population a statistical difference was observed (p < 0.05) in all the dimensions, with a very important impairment in physical function (median: 0; p25–75: 0–10) and physical role (median: 0; p25–75: 0–6.25) (Table 2, Fig. 1). Likewise, in the EQ-5D questionnaire, patients with ALS presented an EQ-5D index score of 0.21 ± 0.39 (mean ± standard deviation) with a visual analog scale (VAS) score of 0.32 ± 0.24. Compared with patients with cancer, patients with ALS had a worse EQ-5D index score both clinically and statistically significant (0.21 ± 0.39 vs. 0.77 ± 0.27; p < 0.05) (Table 2).

**Table 2:** SF-36 and EQ-5D quality of life questionnaires in patients with amyotrophic lateral sclerosis.

SF-36	Cohort study (n = 23)	Population-based reference^10^ (n = 9151)	P value
Physical function (0–100)			< 0.0001
Median (range)	0 (45)	95 (100)	
Mean (standard deviation)	8.7 (13.3)	84.7 (24)	
Role physical (0–100)			< 0.0001
Median (range)	0 (58.3)	100 (100)	
Mean (standard deviation)	10.6 (19.5)	83.2 (35.2)	
Body pain (0–100)			0.0005
Median (range)	42 (88)	100 (100)	
Mean (standard deviation)	49.7 (34.6)	79 (27.9)	
General health (0–100)			< 0.0001
Median (range)	27.5 (40)	72 (100)	
Mean (standard deviation)	29.1 (13.7)	68.3 (22.3)	
Vitality (0–100)			< 0.0001
Median (range)	28.1 (93.8)	70 (100)	
Mean (standard deviation)	33.9 (29.7)	66.9 (22.1)	
Social functioning (0–100)			< 0.0001
Median (range)	25 (100)	100 (100)	
Mean (standard deviation)	37.5 (35)	90.1 (20)	
Role emotional (0–100)			0.0008
Median (range)	50 (100)	100 (100)	
Mean (standard deviation)	56.8 (39.4)	88.6 (30.1)	
Mental health (0–100)			0.001
Median (range)	52.5 (90)	76 (100)	
Mean (standard deviation)	52.9 (27.7)	73.3 (20.1)	

EQ-5D	Cohort study (n = 23)	Cancer population (n = 249)^14^	P value
Index score			0.02
Median (range)	0.12 (1.27)		
Mean (standard deviation)	0.21 (0.39)	0.77 (0.27)	

Comparison of scores of patients with amyotrophic lateral sclerosis versus the general population^10^ and versus patients with cancer^14^.

## Discussion

This prospective study showed that ALS is a neurodegenerative disease that significantly affects quality of life. The quality of life of patients with ALS is statistically and clinically worse than in patients with cancer and worse than the general population. Similar studies have addressed this topic although this impact in quality of life were not compared with other disease. A prospective study conducted in 2017 in 23 patients with ALS^15^ showed that as the disease progresses, the quality of life and functionality of the patients deteriorates. Likewise, a longitudinal prospective study in 36 patients with ALS^14^ showed the importance of motor disability and its relationship with psycho-emotional pathology such as depression and anxiety.

Our work has several strengths. First, we have collected data about all patients evaluated which allows us to identify different characteristics between the patients who entered in the study versus those who did not participate, which adds value to the internal and external validity of the results. Moreover, patients that decided not to participate in QoL study had more reduce mobility and wheelchair, which probably implies worse quality of life what indicates that our results overestimated quality of life in this population. Second, this study compared QoL in patients with ALS with other population (general population and patients with cancer) which allow us to evaluate the real impact of QoL in ALS.

Regarding limitations of the study. First, we acknowledge the limitation of our small sample size when compared to the number of patients recruited in studies conducted on other pathologies. However, it is crucial to consider that ALS is a rare disease, and the number of patients included in our study is proportionate to what is expected in a reference unit in ALS. Second, it would be interesting to gather data from other tests, such as the Edinburgh Cognitive and Behavioural ALS Screen (ECAS)^16^, specific questionnaires to evaluate anxiety or depression, or the Milano-Torino Staging (MiToS)^17^. However, most of these questionnaires are tedious, and patients often become exhausted when completing multiple assessments. We considered the possibility that conducting several tests might discourage patient or family member participation in the study due to the time required. Assessing of these questionnaires and a larger sample size would have allowed us to evaluate variables associated with a worse quality of life. Third, in order to be able to compare several populations, it would have been more appropriate, from a methodological point of view, to evaluate the QoL questionnaires at the same time, and in the context of the same study. Even so, the population with cancer used to compare the QoL came from the same center and was carried out in a similar period.

## Conclusions

Quality of life in patients with ALS is poor, and clinically and statistically worse than in patients with cancer or general population. Several strategies should be implemented improve the quality of life of patients with ALS and new studies that evaluate the impact of each of the measures adopted in this population should be done.

## Supplementary Information

Supplementary Table 1.

## Data Availability

The datasets used and analyzed during the current study are available from the corresponding author on reasonable request.

## Abbreviations

ALS: Amyotrophic lateral sclerosis
VTE: Venous thromboembolism
QoL: Quality of life
EQ-5D: EuroQoL 5D
PE: Pulmonary embolism
EFNS: European Federation of Neurological Societies
HRQoL: Health-related quality of life
FVC: Forzed vital capacity
US: Ultrasonography
V/Q: Ventilation/perfusion
CT: Computerized tomography
ALSFRS-R: Amyotrophic Lateral Sclerosis Functional Rating Scale
SF-36: Short Form-36
UI: Utility index
MID: Minimally important difference
CI: Confidence interval
VAS: Visual analog scale
