# Supplementary material for: Prospective study to evaluate quality of life in amyotrophic lateral sclerosis
Source: Sci Rep. 2023 Jul 26;13:12074. doi: 10.1038/s41598-023-39147-w (PMC10372064; doi:10.1038/s41598-023-39147-w)
Supplement: Supplementary file 1 — Supplementary Table 1. [file 41598_2023_39147_MOESM1_ESM.pdf]

SUPPLEMENTARY MATERIAL

Table 1. Baseline demographics and clinical features of patients with amyotrophic lateral sclerosis comparing patients that completed quality of life questionnaires and those that not.

	**QoL questionnaire NO (n=21)**	**QoL questionnaire SI (n=23)**	**Total (n=44)**
Men, n (%)	10 (47.6)	10 (43.5)	20
Age (years), mean ± DE	58.2±11.8	65.1±12.6	61.8±12.6
Current smoker, n (%)	3 (14.3)	6 (26.1)	9 (20.5)
Familiar ALS, n (%)	3 (14.3)	2 (8.7)	5 (11.4)
Symptoms			
Bulbar, n (%)	7 (33.3)	10 (43.5)	17 (38.6)
Dysphagia, n (%)	8 (38.1)	10 (43.5)	18 (40.9)
Syalorrhea, n (%)	3 (14.3)	2 (9.5)	5 (11.9)
Dyspnea, n (%)	4 (19)	7 (30.4)	11 (25)
Orthopnea, n (%)	3 (14.3)	3 (13)	6 (13.6)
Headache, n (%)	1 (4.8)	3 (13)	4 (9.1)
Hyper-somnolence, n (%)	2 (9.5)	4 (17.4)	6 (13.6)
Reduced mobility, n (%)	16 (80)	14 (60.9)	30 (69.8)
Use of wheelchair, n (%)	14 (66.7)	8 (34.8)	22 (50)
ALSFR, mean ± DE	34.5±11.6	34.3±8	34.4±9.9
Non-invasive ventilation, n (%)	11 (52.4)	6 (26.1)	17 (38.6)
Tracheostomy, n (%)	2 (9.5)	0	2 (4.5)
Physiotherapy, n (%)	17 (85)	18 (78.3)	35 (81.4)
Inspirometer, n (%)	18 (85.7)	18 (78.3)	36 (81.8)
Manual assisted cough, n (%)	9 (42.9)	8 (34.8)	17 (38.6)
Cough Assist, n (%)	4 (21.1)	0 (0)	4 (9.8)
Oxygen therapy, n (%)	7 (33.3)	2 (9.1)	9 (20.9)
Percutaneous gastrostomy, n (%)	4 (19)	3 (13)	7 (15.9)
**First evaluation in respiratory unit**			
FVC (%),mean ± DE	91.8±25.1	92±19.3	91.9±22.8
pCO2 (mmHg), mean ± DE	46.1±15.8	39.9±4.7	42.3±10.5
pO2 (mmHg), mean ± DE	77.8±20.9	77.1±10	77.4±14.9
Initial peak flow, mean ± DE	331.8±141.3	305.5±121	319.6±131.3
**At the start of non-invasive ventilation**			
FVC (%), mean ± DE	61.4±20.4	52.3±6.1	57.3±15.7
pCO2 (mmHg), mean ± DE	55.5±16.2	52±7.1	53.8±11.7
pO2 (mmHg), mean ± DE	67.5±20.9	77.1±10	77.4±14.9
HCO3 (mmol/l), mean ± DE	32.5±7.3	29.1±5.5	31±6.3

QOL: Quality of Life; ALS: Amyotrophic Lateral Sclerosis; ALSFS: Amyotrophic Lateral Sclerosis Functional Rating; FVC: Forced vital capacity.
